# Stimulus control of habits: Evidence for both stimulus specificity and devaluation insensitivity in a dual‐response task

**DOI:** 10.1002/jeab.898

**Published:** 2023-12-15

**Authors:** K. M. Turner, B. W. Balleine

**Affiliations:** ^1^ School of Psychology University of New South Wales Sydney Australia

**Keywords:** devaluation, discriminative stimulus, habits, rats, stimulus control

## Abstract

Goal‐directed and habitual actions are clearly defined by their associative relations. Whereas goal‐directed control can be confirmed via tests of outcome devaluation and contingency‐degradation sensitivity, a comparable criterion for positively detecting habits has not been established. To confirm habitual responding, a test of control by the stimulus–response association is required while also ruling out goal‐directed control. Here we describe an approach to developing such a test in rats using two discriminative stimuli that set the occasion for two different responses that then earn the same outcome. Performance was insensitive to outcome devaluation and showed stimulus–response specificity, indicative of stimulus‐controlled behavior. The reliance of stimulus–response associations was further supported by a lack of sensitivity during the single extinction test session used here. These results demonstrate that two concurrently trained responses can come under habitual control when they share a common outcome. By reducing the ability of one stimulus to signal its corresponding response–outcome association, we found evidence for goal‐directed control that can be dissociated from habits. Overall, these experiments provide evidence that tests assessing specific stimulus–response associations can be used to investigate habits.

## INTRODUCTION

There is now considerable evidence that the performance of instrumental actions can be mediated by two forms of action control, one more flexible and goal directed and the other more reflexive and habitual (Balleine & Dezfouli, 2019). These differences in control have been argued to emerge from distinct associative structures, with goal‐directed actions mediated by response–outcome (R‐O) associations and habits by stimulus–response (S‐R) associations (Balleine & Dickinson, 1998; Dickinson & Balleine, 1994). These associative structures result in distinct patterns of behavior: when under goal‐directed control, instrumental actions are sensitive to changes in both the R‐O relationship and the value of O; whereas, when under habitual control, actions are insensitive to both of these manipulations (Balleine & Dickinson, 1998; Dickinson, 1985; Dickinson & Balleine, 1994).

These behavioral differences have become defining features of these actions and are often used to identify which form of control is currently governing a response. However, although these behavioral effects provide positive criteria with which to identify goal‐directed actions, as a definition of habits they are much less convincing. Although using *insensitivity* to degradation and devaluation treatments to define a habit has become commonplace, it relies on affirming the null hypothesis (i.e., the contingency and value changes have no effect relative to controls) and risks asserting habitual control when animals are merely confused or when goal‐directed control is impaired (Balleine & Dezfouli, 2019; Watson et al., 2022). This issue has been increasingly recognized, and in recent years there have been attempts to improve the definition of habits through a focus on their rapid execution, inflexibility, and relationship to the formation of chunked action sequences (Dezfouli & Balleine, 2012; Garr & Delamater, 2019; Thrailkill et al., 2021; Turner et al., 2022; van Elzelingen et al., 2022; Vandaele & Janak, 2021; Vandaele et al., 2017). These attempts have helped to document important features of habits that were not directly captured by more traditional models. However, an agreed set of criteria allowing habits to be directly observed and discriminated from other influences on instrumental performance has failed to emerge.

One reason for this failure has been a general reluctance to interrogate the nature of the S‐R associations from which habits are built. This has led researchers to question whether there is any direct evidence for S‐R associations in rodents at all (Vandaele et al., 2017). Indeed, although hierarchical frameworks have been developed that have incorporated the contribution of the stimulus, response, and outcome, few studies have reported evidence for or against different possible combinations of these events (Balleine & Dezfouli, 2019; Bradfield & Balleine, 2013; Colwill & Rescorla, 1990b; Rescorla, 1991). Early demonstrations of habit used situations in which the control of a response by a specific stimulus was merely assumed or implicit. More recently, however, studies have accumulated in which explicit discriminative stimuli have been used to control the response and that have demonstrated the importance of consistent reinforcement of a response within a stimulus for generating habits (Faure et al., 2005, 2010; Steinfeld & Bouton, 2020; Thrailkill et al., 2018; Vandaele et al., 2017). For example, Thrailkill and colleagues found that when consistently reinforced during a stimulus, the response was insensitive to outcome devaluation, whereas when reinforced during 50% of stimulus presentations responding becomes sensitive to devaluation (Thrailkill et al., 2018, 2021).

These demonstrations of stimulus control have the potential to identify habits, particularly if the specificity of that control could be readily demonstrated. However previous attempts to incorporate two responses in the same session have failed to generate devaluation insensitivity (Kosaki & Dickinson, 2010; Shipman et al., 2018; Trask et al., 2020), and, indeed, reinforcing different responses under distinct discriminative stimuli often causes goal‐directed control to be maintained, even after significant overtraining (Colwill & Rescorla, 1990a, 1990b; Trask et al., 2020). Nevertheless, many of these attempts were not optimized for the development of habits, often using both distinct reinforcers and distinct actions without ensuring consistent reinforcement of the responses under the stimuli. For example, using an elegant design that balanced binary associations, Colwill and Rescorla (1990b) trained rats on two different responses that earned two different rewards, where the stimulus discriminated the specific relation between them (i.e., R1 was associated with O1 under S1 but O2 under S2). In a subsequent devaluation test, the rats were able to use the current stimulus to determine which response to make to earn the still valued outcome, providing compelling evidence for hierarchical S‐(R‐O) associations. Here we sought to modify this approach to establish two distinct S‐R associations that earned the same outcome in the hope that these associations would generate distinct S‐R habits insensitive to devaluation but under clear stimulus control.

Therefore, in experiment 1 we set out to determine whether dissociable S‐R associations could be developed using distinct discriminative stimuli if the responses earned the same outcome (Colwill & Rescorla, 1990b; Thrailkill et al., 2018; Trask et al., 2020). To provide evidence for this specificity, we assessed both the degree of habitual performance for each response, using outcome devaluation, and the extent to which the distinct stimuli exerted control over the different responses. In the face of evidence that a response is insensitive to outcome devaluation, satisfying this *response‐specificity criterion* would allow us to establish the degree to which each habit was maintained by specific S‐R control. In Experiment 2 we sought to restore goal‐directed control for one response by altering the predictability of its associated stimulus while maintaining the predictability of the other stimulus to determine whether both goal‐directed and habitual control can be simultaneously maintained or whether one form of control would dominate both responses.

## METHODS

### Subjects

Male and female adult Long Evans rats (UNSW colony, Australia) were housed in groups of four in a climate‐controlled vivarium (12‐hr light cycle, lights on 0600 hours). Each experiment consisted of mixed sex cohorts (Experiment 1: 11 males, 9 females; Experiment 2: 9 males, 14 females). The rats received a tunnel and wood block in individually ventilated cages, with corn cob bedding and free access to water throughout the experiment. The rats were food restricted to ~90%–95% free‐feeding body weight and handled for 3 days prior to training and throughout the experiment. All procedures were approved by the UNSW Animal Care and Ethics Committee and followed the Australian Code for the Use and Care of Animals (NHMRC).

### Apparatus

Standard Med Associates (Vermont, USA) rat operant chambers were fitted with a central magazine, with two horizontal levers positioned to its left and right on one wall. On the opposite wall, the boxes also contained a clicker (28 V), a houselight (3 W, 28 V), and a white noise generator (70 dB). The magazine was connected to a pellet dispenser that delivered 45‐mg sucrose pellets (BioServ, USA). Each chamber was fitted with an overhead camera for monitoring behavior and positioned within sound‐attenuating chambers.

### Procedure

#### Experiment 1: Instrumental training

**FIGURE 1:**
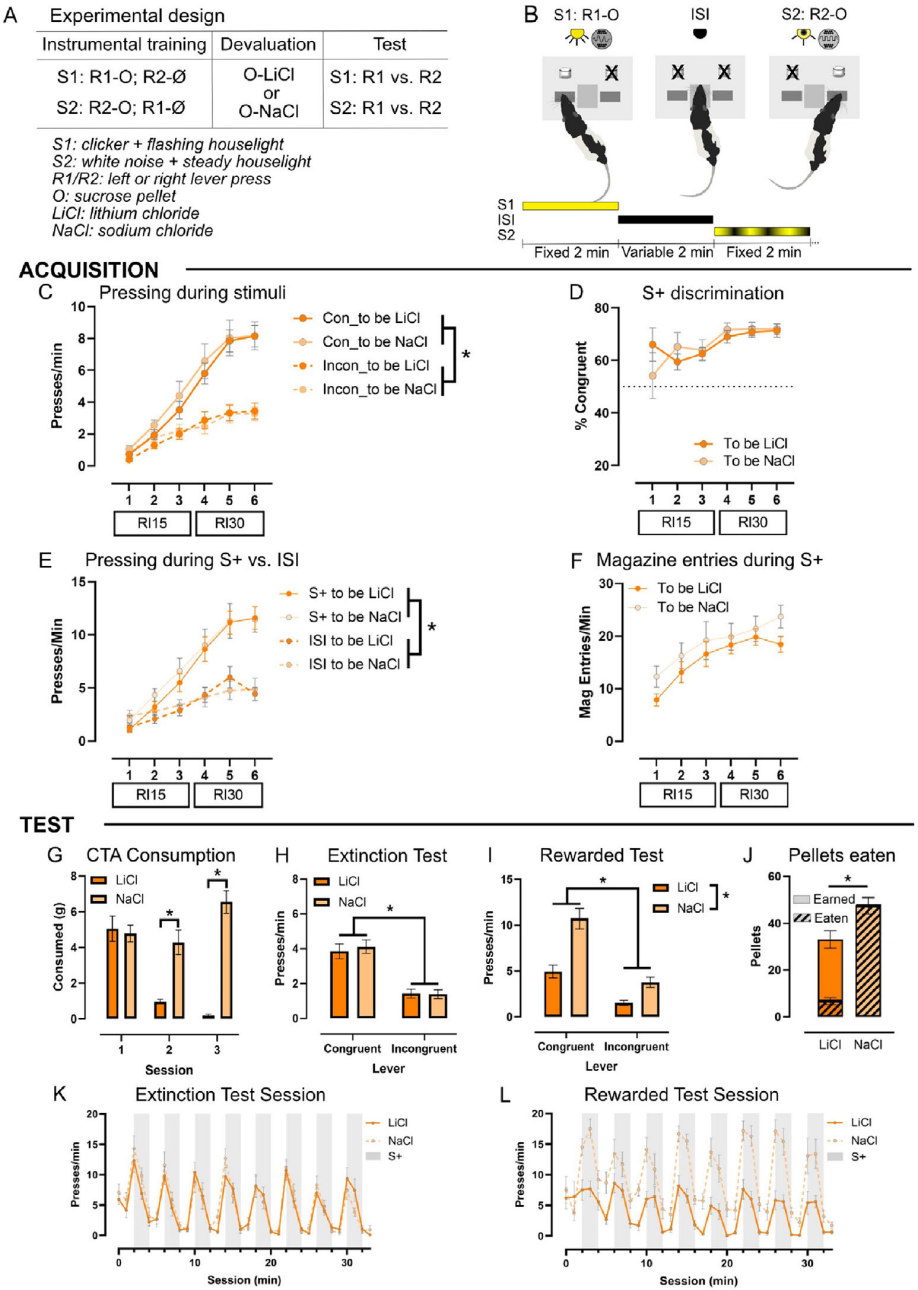
Habitual responding on two concurrent responses using discriminative stimuli to identify specific S‐R associations. (A) stages of training and devaluation testing, (B) experiment diagram, (C) lever‐press rates on the congruent (con) and incongruent (incon) levers during training in groups that will be devalued (LiCl) and valued (NaCl) before test, and (D) percentage of presses that were on the congruent lever during training in each group. (E) the rate of lever presses during the discriminative stimuli (S+) increased relative to that during the interstimulus interval (ISI) across training in both groups; (F) the rate of magazine entries during the discriminative stimuli also increased with training but did not differ between groups; (G) the devalued group (LiCl) showed conditioned taste aversion via a significantly reduced consumption of reward pellets by the third conditioning session; and (H) lever‐pressing rates in the extinction test did not significantly differ between LiCl‐ and NaCl‐treated rats, indicating responding was not sensitive to outcome devaluation. In addition, rats responded significantly more on the congruent than incongruent lever, demonstrating expression of the specific stimulus–response associations learned during training; (I) the control group (NaCl) group pressed significantly more than the devalued group (LiCl) group on the rewarded test, demonstrating that in the presence of the outcome taste aversion in the devalued group was sufficient to reduce responding and (J) reduced the number of earned pellets earned and consumed. Across the (K) extinction test session, both groups pressed more during the S+ than the ISI and more in the first than the second minute of the S+. Rats pressed more in the first block but then did not decrease pressing across the rest of the session. (L) In the rewarded test, again all the rats pressed more in the S+ than the ISI, and more in the first than the second minute of the S+. The NaCl rats pressed more than the LiCl rats, except in the first ISI block, which was before any rewards were delivered. *p < .05; mean ± SEM, *n* = 10/group.

The experimental design is illustrated in Figure 1A and B. Rats first received one ~40‐min session of magazine training in which 40 sucrose pellets were delivered on a variable time (VT) 60 schedule. This was followed by one session of lever‐press training on a continuous reinforcement schedule. Both levers were extended from the start of the session, and each lever press was rewarded until the rats had obtained 20 outcomes per lever, with each lever retracting once the maximum was achieved or 40 min had elapsed.

The rats were then trained on escalating random‐interval (RI) schedules using the discriminative stimuli to signal which response was reinforced. The discriminative stimuli (S1, S2) were audiovisual compounds consisting of either background noise and steady houselight, or 10‐Hz clicker with the houselight flashing at 10 Hz. Audiovisual compounds were used to ensure stimuli could be easily discriminated. Sessions consisted of 12 presentations of S1 and S2 (six of each in pseudorandom order that changed each session) that were 2 min in duration and separated by variable 2‐min interstimulus intervals (ISI; range: 1–3 min). Both levers were extended at all times, and each 2‐min stimulus presentation is described as a stimulus block. The rats were rewarded on RI schedules when they pressed on the lever that was congruent with the stimulus being presented (i.e., R1 during S1, R2 during S2). The congruent response in each stimulus earned the same sucrose pellet outcome. Presses on the incongruent lever during the S+ presentations (e.g., R2 during S1, R1 during S2) and lever presses during ISI periods were not rewarded. Sucrose pellets were delivered on an RI15 schedule for three sessions before moving to RI30 for the next three sessions. Therefore, there were a total of six RI training sessions. Stimulus–response pairings were counterbalanced across the cohort but constant for each rat (see Figure 1A and B).

#### Experiment 1: Outcome devaluation by conditioned taste aversion

The rats were allocated to the valued (NaCl) or devalued (LiCl) group, matched for performance on the final RI30 session based on press rate, outcomes earned, congruency, and magazine entries. They were placed in individual empty Techniplast mouse cages with wire top lids in a dimly lit quiet room and given 15 g of sucrose pellets for 30 min. They were removed and immediately injected (i.p.; 20 mL/kg) with either lithium chloride (LiCl, 0.15 M) in the devalued group or sodium chloride (NaCl, 0.15 M) in the valued group. The rats were immediately returned to their home cage. This was repeated over 3 days, and the number of pellets that was consumed each day was recorded.

#### Experiment 1: Extinction and rewarded tests

The rats were then tested in extinction using eight 2‐min stimulus presentations, each separated by a fixed 2‐min ISI period (counterbalanced across cohort using an A‐B‐B‐A‐B‐A‐A‐B order). They were then given a rewarded test session using the same stimulus presentations as in extinction except that sucrose pellets were delivered on an FI15 schedule. This test was conducted to confirm that, in the presence of the outcome, the devalued group reduced responding relative to the valued group. The number of earned pellets left uneaten at the end of the rewarded session was also recorded.

#### Experiment 2: Stimulus contingency manipulation

**FIGURE 2:**
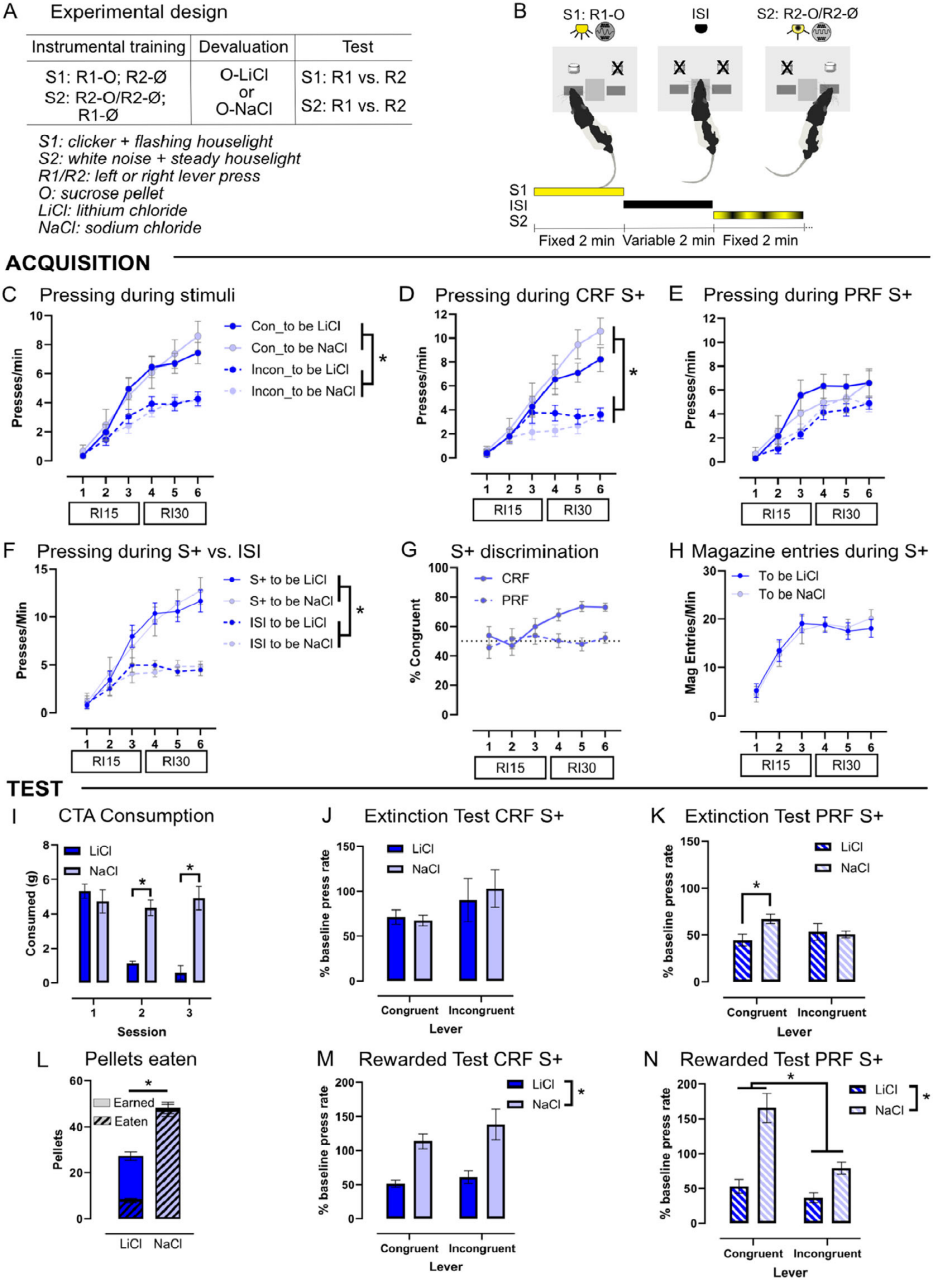
Partial nonreinforcement leads to goal‐directed control. (A) stages of training and devaluation testing, (B) experimental design, and (C) lever press rates during training. (D) press rates on the congruent lever increased more than on the incongruent lever with training in the CRF condition; (E) press rates on the congruent lever were not as clearly increased over the incongruent lever with training in the PRF condition; (F) lever pressing increased during the S+ vs. ISI across training; (G) discrimination increased across training under the CRF but not PRF stimuli; (H) magazine entries also increased across training and none of the training variables differed for the “to‐be” LiCl and NaCl treatment groups; (I) rats given LiCl significantly reduced consumption of reward pellets by the third conditioning session; (J) in the extinction test, the LiCl and NaCl groups did not differ in % baseline press rates during the CRF stimuli, demonstrating devaluation insensitivity; (K) however, under the PRF stimuli the LiCl group pressing significantly less than the NaCl group on the congruent lever, indicating rats were sensitive to outcome devaluation when the stimulus was less predictive. (L) On the rewarded test, the number of pellets earned and consumed was significantly less in the LiCl group than in the NaCl group. (M) In the rewarded test NaCl group pressed significantly more than the LiCl group during the CRF and (N) PRF stimuli, demonstrating that taste aversion in the LiCl group was sufficient to reduce responding. *p < .05, mean ± SEM, *n* = 12/group.

Experiment 2 was identical to Experiment 1 except for the final three RI30 sessions. For the final three sessions, one stimulus (e.g., S1+) was rewarded based on an RI30 schedule, but only on 50% of the presentation blocks with the other 50% of presentations conducted in extinction. During the other stimulus (e.g., S2+), the congruent lever continued to be rewarded on an RI30 schedule during every stimulus presentation. Previously, there was no limit on the number of outcomes that could be earned within the 2‐min stimulus windows; however in Experiment 2 there was an eight‐outcome limit (based on RI30 over 2 min) imposed on the always‐reinforced S+ to reduce excessive imbalance of reinforcement across the levers by chance (see Figure 2A and B).

### Statistical analysis

There were no sex differences detected (all *p*s > .30). Consequently, all of the analyses were pooled across sex. To confirm that the rats learned the discrimination, the lever‐press rates on the congruent versus incongruent responses were calculated as percentage of congruent presses: congruent/(congruent + incongruent). Between‐subjects and repeated‐measures analyses of variance (ANOVAs) were used to compare lever pressing, percentage of congruent presses, and magazine entries during training for the various groups. The values for pellets consumed during taste‐aversion conditioning were compared for the valued and devalued groups. For the extinction and rewarded tests in Experiment 1, the press rates on the congruent and incongruent levers were analyzed by using 2 × 2 mixed‐model ANOVAs (between subjects: devaluation group, within subjects: lever congruency).

In Experiment 2, the effect of consistent (CRF) versus inconsistent (PRF) reinforcement of the two responses was included as a factor. Although devaluation groups were not significantly different during training, the group means were not identical. Therefore, devaluation sensitivity was examined using the percentage of baseline press rates, calculated based on the final two training sessions. Baseline correction normalizes the effect of congruency, so this factor was assessed using press rates as in Experiment 1. For each ANOVA, post hoc comparisons with the Bonferroni correction were conducted when a significant interaction was detected (SPSS Inc., USA). Differences were considered statistically significant at α = .05 for all analyses.

## RESULTS

### Experiment 1: Discriminative stimulus‐driven habits

#### Task acquisition

Figures 1C–F illustrate task acquisition showing that rats increased their press rate across training, *F*_(2,33)_ = 79.9, *p* < .001, particularly on the congruent lever as shown by a repeated‐measures ANOVA: lever, *F*_(1,18)_ = 115.29, *p* < .001; Session × Lever, *F*_(2,42)_ = 51.57, *p* < .001 (Figure 1C). There was no main effect or interactions detected with the groups to be devalued versus not devalued (to‐be LiCl vs. to‐be NaCl injected; *p*s > .50). When comparing the percentages of congruent presses for the “to‐be” devalued groups, there was no main effect of group, *F*_(1,18)_ < 0.001, *p* = .99, or interaction with training session, *F*_(5,90)_ = 1.12, *p* = .36 (Figure 1D). When comparing presses during the stimulus and ISI windows, there was no main effect of “to‐be” devalued group, *F*_(1,18)_ = 0.18, *p* = .68, or interactions between the devaluation conditions (*p*s > .34). However, there were significantly more presses made during the S+ than during the ISI, *F*_(1,18)_ = 84.10, *p* < .001, and this dissociation strengthened across training as shown by an interaction with session: *F*_(2,33)_ = 70.14, *p* < .001 (Figure 1E). Magazine entries during the S+ also increased across training, *F*_(3,48)_ = 20.36, *p* < .001, but did not differ by devaluation conditions (main effect and interaction: *p*s > .30; Figure 1F). Generally, therefore, we found strong evidence for control of instrumental responses by their respective discriminative stimuli.

#### Outcome devaluation: Conditioned taste aversion

Over the course of devaluation, sucrose pellet consumption was significantly reduced in the LiCl‐ vs. the NaCl‐treated rats as shown by a repeated‐measures ANOVA: main effect day, *F*_(2,36)_ = 13.25, *p* < .001; main effect of devaluation, *F*_(1,18)_ = 36.84, *p* < .001; and Day × Devaluation, *F*_(2,36)_ = 26.53, *p* < .001 (Figure 1G).

#### Outcome devaluation: Extinction and rewarded tests

The main data of interest are those from the devaluation extinction test, summarized in Figure 1H. In this test, both LiCl‐ and NaCl‐treated rats pressed more on the congruent than incongruent lever and to a similar extent, indicating there was no effect of devaluation, repeated‐measures ANOVA: main effect of lever, *F*_(2,36)_ = 194.32, *p* < .001; main effect of devaluation and interaction, *p*s > .40 (Figure 1H). Therefore, the rats were able to express the specific response associated with each stimulus but were insensitive to outcome devaluation.

In the rewarded test, rats in both conditions again pressed more on the congruent than the incongruent lever. However, in contrast to the extinction test, the NaCl‐treated rats pressed more than the LiCl‐treated rats did and there was a significant interaction, repeated‐measures ANOVA: main effect of lever, *F*_(1,18)_ = 116.54, *p* < .001; main effect of devaluation, *F*_(1,18)_ = 19.31, *p* < .001; and interaction, *F*_(1,18)_ = 13.39, *p* = .002 (Figure 1I). The LiCl‐treated rats showed significant discrimination performance (mean difference = 3.43, *p* < .001), but this was lower than it was for the NaCl‐treated rats (mean difference = 6.95, *p* < .001), consistent with the overall reduction in pressing. This result confirmed outcome devaluation in the LiCl‐treated group. They also earned, *t*(18) = 8.5, *p* < .009, and consumed fewer of the pellets that were earned on the rewarded test, *t*(18) = 117.3, *p* < .001 (Figure 1J).

We also investigated changes within the extinction and rewarded tests by examining press rates across the eight S+ and ISI blocks in both groups in one‐minute bins to assess both within‐ and between‐block effects on presses during the stimulus and interval periods (Figure 1K). In the extinction test, there was a significant effect of block type, with higher press rates in the S+ than in the ISI blocks, *F*_(1,18)_ = 128.58, *p* < .001). There was also a main effect of block, *F*_(7,126)_ = 12.70, *p* < .001), indicating that the rats pressed more in the first blocks but then continued to press at the same rate across the next seven S+ and ISI blocks (Bonferroni corrected post hoc: *p*s > .40 with all other blocks) but then continued to press at the same rate across the next seven S+ and ISI blocks. However, within each 2‐min stimulus block there was a significant drop in pressing during the second minute relative to the first minute that was not detected in ISI blocks: Block Type × Minute, *F*_(1,18)_ = 43.13, *p* < .001; ISI 1st vs. 2nd min *p* = .51; S+ 1st vs. 2nd *p* < .001. There was no main effect (*p* = .70) or interactions with devaluation treatment (*p*s > .09). These results showed that the rats consistently reduced pressing during the stimulus presentation (i.e., from the first to second minute) but continued to press at each S+ presentation throughout the session, showing no additional extinction of responding beyond the first blocks (Figure 1K).

In the rewarded test, shown in Figure 1L, there was a main effect of group such that the NaCl‐treated rats pressed more than the LiCl‐treated rats, *F*_(1,18)_ = 19.31, *p* < .001, as well as main effects of block type, S+ vs. ISI, *F*_(1,18)_ = 105.03, *p* < .001; minute, *F*_(1,18)_ = 7.35, *p* = .014; and block, *F*_(4,65)_ = 4.97, *p* = .002. Overall, this indicated that the rats pressed more during the S+ than the ISI blocks and more in the first minute than in the second minute. There was a significant interaction between devaluation group, block type, and block, *F*_(7,126)_ = 3.18, *p* = .004, indicating that the devaluation groups differed in press rates in all blocks except for the first ISI block, which was prior to the first reward delivery (*p* = .79; Figure 1L).

### Experiment 2: Partially reinforced discriminative stimulus

#### Task acquisition

The design and illustration of the task used in Experiment 2 are presented in Figure 2A and B, respectively, and the data from the acquisition of the discrimination in Experiment 2 is summarized in Figures 2C–H. Lever pressing increased across training as rats learned to press on the congruent lever, as shown by a repeated‐measures ANOVA: session, *F*_(3,66)_ = 84.08, *p* < .001; lever, *F*_(1,21)_ = 38.86, *p* < .001; Session × Lever, *F*_(5,105)_ = 36.93, *p* < .001 (Figure 2C). There were no significant main effects or interactions involving the devaluation conditions (LiCl vs. NaCl groups; *p*s > .40). Press rates for the CRF and PRF lever were then analyzed separately. The result for CRF resembled the overall results, with a main effect of session and lever as well as a significant interaction, *F*_(3,57)_ = 26.56, *p* < .001 (Figure 2D). The results for the PRF stimulus indicated that the rats did increase lever pressing across training, *F*_(3,60)_ = 64.27, *p* < .001, and showed a smaller but still significant bias toward selecting the congruent over the incongruent lever, *F*_(1,21)_ = 4.68, *p* = .042. There was a Session × Lever interaction, *F*_(3,70)_ = 3.50, *p* = .016, with congruent press rate being significantly greater than incongruent press rate on the third session of RI15 and the final session of RI30 under the PRF stimulus (Figure 2E). There was no main effect or interactions between these measures and the devaluation conditions, confirming the LiCl and NaCl groups were matched for performance.

Overall, the rats pressed more during the stimulus than in the ISI blocks, *F*_(1,21)_ = 70.54, *p* < .001, and this effect increased with training: interaction, *F*_(2,48)_ = 66.55, *p* < .001 (Figure 2F), without being different for the devaluation groups (*p*s > .40). Discrimination significantly increased with training during the CRF stimuli but not the PRF stimuli, repeated‐measures ANOVA: Lever × Interval, *F*_(3,58)_ = 8.01, *p* = .031 (Figure 2G), but again no significant main effect or interaction with the devaluation group allocation was found (*p*s > .20). Magazine entries during the S+ blocks across training increased, with a significant effect of session, *F*_(3,67)_ = 35.59, *p* < .001 (Figure 2H), also with no significant main effect or interaction with “to‐be” devaluation group (*p*s > .80).

#### Outcome devaluation: Conditioned taste aversion

Pellet consumption was significantly reduced in the LiCl‐ relative to the NaCl‐treated rats over the 3 days of injections, repeated‐measures ANOVA: main effect day, *F*_(2,42)_ = 39.73, *p* < .001; main effect of devaluation, *F*_(1,21)_ = 20.66, *p* < .001; and Day × Devaluation, *F*_(2,42)_ = 21.33, *p* < .001 (Figure 2I).

#### Outcome devaluation: Extinction and rewarded tests

In the extinction test, the rats pressed the congruent lever significantly more than the incongruent lever during the CRF stimulus blocks, *F*_(1,21)_ = 68.82, *p* < .001. Reducing the predictive accuracy of the PRF stimulus also produced a significant bias toward the congruent over the incongruent lever, *F*_(1,21)_ = 6.53, *p* = .018, although the effect was not as large as during the CRF stimulus. Press rates during the PRF stimuli were lower, however a comparison with ISI press rates confirmed this was not a floor effect, as rats pressed at a significantly lower rate during the ISI than during either the valued or devalued conditions under the CRF or PRF stimuli: main effect, *F*_(4,92)_ = 35.70, *p* < .001 (*p* < .05 for all four comparisons). Devaluation sensitivity was assessed as the percentage of baseline responding during the CRF and PRF stimuli to account for differences in press rate during training. A repeated‐measures ANOVA found there was no effect of devaluation or interaction with congruency (*p*s > .10) during the CRF stimuli (Figure 2J). However, during the PRF stimuli there was a significant Devaluation × Congruency interaction, *F*_(1,21)_ = 10.12, *p* = .005. This result indicated that rats in the valued group responded significantly more than those in the devalued group on the congruent lever (*p* = .010) but not the incongruent lever (*p* = .77; Figure 2K). This result was consistent with our hypothesis that the PRF condition would restore devaluation sensitivity.

On the rewarded test, the rats pressed the congruent lever significantly more than the incongruent lever and demonstrated reduced responding in the devalued group during the CRF stimulus: main effect lever, *F*_(1,21)_ = 56.21, *p* < .001; devaluation, *F*_(1,21)_ = 43.03, *p* < .001; Lever × Devaluation, *F*_(1,21)_ = 19.44, *p* < .001. The significant interaction indicated the effect of devaluation was greater on the congruent than the incongruent response; however, a significant effect of devaluation was detected on all post hoc comparisons (*p*s < .02). Similar results were found under the PRF stimulus: main effect lever, *F*_(1,21)_ = 26.48, *p* < .001; devaluation, *F*_(1,21)_ = 12.21, *p* = .002; Lever × Devaluation, *F*_(1,21)_ = 3.94, *p* = .060. Devaluation was also assessed as a percentage of baseline responding, which revealed a main effect of devaluation under the CRF, *F*_(1,21)_ = 23.34, *p* < .001 (Figure 2M) and PRF conditions, *F*_(1,21)_ = 11.77, *p* = .003 (Figure 2N). There was also a main effect of congruency detected under the PRF stimuli, *F*_(1,20)_ = 25.54, *p* < .001, with rats pressing at higher rates than baseline on the congruent lever: interaction, *F*_(1,21)_ = 11.77, *p* = .003. This is not surprising given that this stimulus was now reinforced on every occurrence, unlike the final session of training. LiCl‐treated rats pressed less on the rewarded test so earned fewer pellets, *t*(21) = 6.85, *p* < .001 (Figure 2L) but also consumed a smaller proportion of the pellets earned than did the NaCl‐treated rats, *t*(21) = 17.49, *p* < .001. This result demonstrated that outcome devaluation was successful given that it significantly reduced responding and outcome consumption in the LiCl‐treated rats.

## DISCUSSION

Habits, defined as instrumental actions that are controlled by S‐R associations, are now commonly identified by the absence of goal‐directed control (Balleine & Dezfouli, 2019). The description of habits has been broadened to include features such as rapid execution, invariant topography, and chunking of action sequences in addition to the more traditional criteria of insensitivity to changes in response–outcome contingency or outcome value (Balleine & Dezfouli, 2019). Yet, a positive measure or criterion for control by the S‐R association has remained elusive. In this study, two discriminative stimuli were associated with the reinforcement of two different responses. Critically, and in contrast to previous studies (Bradfield & Balleine, 2013; Colwill & Rescorla, 1990b; Kosaki & Dickinson, 2010), both responses were reinforced with the same outcome. Training under this protocol produced specific stimulus‐controlled responses that were insensitive to outcome devaluation. Using these two measures (devaluation insensitivity and stimulus specificity of control), Experiment 1 confirmed both that responding was not under goal‐directed control and that it was driven by its association with the specific stimulus in which it was reinforced. In addition, we found that in the presence of the discriminative stimulus, responding was relatively insensitive to extinction. Although consistent with a reduced contribution by the outcome to ongoing performance, confirming this effect is specific to habits will require comparison with a goal‐directed control. To our knowledge, this is the first study to demonstrate habitual responding in a task employing two concurrent responses. Habits are typically studied using single‐response tasks because the inclusion of a second intermixed response earning a second outcome has been found in multiple studies to be sufficient to restore goal‐directed control (Bradfield & Balleine, 2013; Colwill & Rescorla, 1990b; Kosaki & Dickinson, 2010; Trask et al., 2020). Here we have demonstrated that when the outcome for both responses is the same, habits can form across two concurrently trained responses.

Experiment 2 assessed whether goal‐directed control could be restored by degrading the predictive relation between the stimulus and the ability to earn the outcome to 50%. In this situation, discrimination of the stimulus–response association was reduced and rats were sensitive to outcome devaluation on the congruent lever. This restoration of goal‐directed control was consistent with the results of prior studies in which devaluation sensitivity was observed after PRF but not CRF manipulations in separate groups of rats that were trained on a single lever (Thrailkill et al., 2018, 2021). Consistent with Experiment 1, outcome devaluation insensitivity was observed under the CRF stimulus. This finding provides further evidence that control over responses is dependent on training conditions (Bouton, 2021; Trask et al., 2020). There are many possible explanations for why PRF responding may have come under goal‐directed control. For example, impaired predictive accuracy of the PRF stimulus could have increased the incorporation of outcome information. Alternatively, or perhaps additionally, PRF extinction blocks may have caused the rats to increase attention to the response–outcome contingency, as might be predicted by recent theoretical approaches (Perez & Dickinson, 2020). There is considerable evidence that goal‐directed control is strengthened when two actions are trained concurrently; however, given our results in Experiment 1, this alone does not lead to devaluation sensitivity. It has previously been demonstrated that changing the reward magnitude and changing the random‐interval schedule are sufficient to restore goal‐directed control under conditions that would otherwise generate habits (Nelson, 2006). However, determining how goal‐directed and habitual responses interact across two responses has been almost impossible given that habits have previously only been generated in single‐response tasks. Nevertheless, given the role of these systems in real‐world examples, dynamic switching between controllers should readily occur, an issue that the current tasks might be developed to study. Further experiments will be needed to address these possibilities.

During the extinction test in Experiment 2, the fact that only the congruent response was sensitive to devaluation is consistent with a presumed associative structure involving the outcome (e.g., S1‐R1‐O) as opposed to the incongruent response (S1‐R2‐Ø). The reliance on discriminative stimuli means binary explanations for the results of Experiment 2 are insufficient. Both the stimuli and responses were equally associated with the outcome, leaving only the S‐R associations as the unique binary association. Nevertheless, the rats were able to respond according to the discriminative stimulus while also being sensitive to outcome devaluation. Here, goal‐directed control appears to require a hierarchical explanation to account for the contribution of associations between stimulus, response, and the outcome. In light of these results, it is interesting to reflect on previous suggestions for the associative structure supporting hierarchical control (Rescorla, 1991). From this perspective, the stimulus is not associated with a specific response but with a specific response–outcome association (S‐[R‐O]). Importantly, the stimulus is not associated with either the response (R) or the outcome (O) in a binary relationship but acts in similar fashion to an occasion setter to select a specific R‐O association over others. A hierarchical associative structure appears more likely than a binary structure to be controlling performance on the task used in Experiment 2, although how control shifts between S‐R and S‐(R‐O) associations when goal‐directed control is reengaged remains to be determined. Neural manipulations, such as those used by Bradfield and Balleine (2013), could be particularly revealing because distinct predictions could be made about the relative contributions of goal‐directed and habitual circuitry during performance of the tasks used here, particularly comparing the effects in Experiments 1 and 2.

Given the novelty of these findings, there are limitations and outstanding questions that need to be addressed in future studies. In this study outcome devaluation compared groups for which outcome consumption was followed by either lithium chloride (devalued group) or saline (valued group). Other designs in which all rats receive injections of lithium chloride, such as those where nausea is explicitly paired or unpaired with outcome consumption or where the reinforced outcome is compared with a novel outcome, could be used to control for the induction of nausea per se (Bouton et al., 2020). Moreover, it would be important to consider the environment used for outcome consumption prior to injections (Bouton et al., 2021). Another consideration worth discussing is whether the same features of the outcome that were used to guide actions were also targeted by outcome devaluation (Delamater & Oakeshott, 2007). For example, the specific taste of the outcome may have been associated with taste aversion, whereas operant responding may be supported by other features such as the outcome's ability to reduce hunger. In essence, the specific features of the outcome that supported responding may not have been the same features associated with devaluation, which could have led to the incorrect assumption that outcome information was not being used when devaluation insensitivity was observed. This will be a challenging problem for future studies to investigate given the numerous features that could be encoded, and identifying specific features incorporated in the instrumental association is not trivial.

In conclusion, we have shown that two concurrently trained instrumental responses can become habitual when their reinforcement is confined to distinct discriminative stimuli, finding that the responses were insensitive to outcome devaluation and showed specificity of stimulus control. Thus, we provide evidence for a loss of goal‐directed control as well as positive evidence that these habits were control by specific S‐R associations. These habitual responses were resistant to extinction, further supporting the claim that the response was under stimulus control. In addition, we found that reducing the predictability of one stimulus was sufficient to restore goal‐directed control, whereas the alternative response remained insensitive. Therefore, we describe a task that is capable of detecting habits and goal‐directed actions, which provides an important step toward identifying the source of action control, particularly in the face of the decision‐making deficits that accompany many neuropsychological disorders.

## FUNDING INFORMATION

KMT was supported by a NARSAD Young Investigator Grant from the Brain and Behavior Research Foundation. BWB received funding from the Australian Research Council (DP200103401), and an NHMRC Senior Investigator Award (GNT1175420).

## CONFLICT OF INTEREST STATEMENT

The authors have no conflicts of interest to declare.

## ETHICS APPROVAL

All procedures were approved by the UNSW Animal Care and Ethics Committee and followed the Australian Code for the Use and Care of Animals (NHMRC).
